# Immediate Effect of Compression Contrast Therapy on Quadriceps Femoris Muscles’ Regeneration in MMA Fighters

**DOI:** 10.3390/jcm13237292

**Published:** 2024-11-30

**Authors:** Robert Trybulski, Robert Roczniok, Kamil Gałęziok, Filip Matuszczyk, Marta Bichowska-Pawęska, Michał Wilk, Jarosław Muracki

**Affiliations:** 1 Medical Department, The Wojciech Korfanty Upper Silesian Academy, 40-659 Katowice, Poland; 2 Provita Żory Medical Center, 44-240 Żory, Poland; 3 Institute of Sport Science, The Jerzy Kukuczka Academy of Physical Education, 40-065 Katowice, Poland; 4 Faculty of Physical Education, Gdansk University of Physical Education and Sport, 80-336 Gdansk, Poland; 5 Institute of Physical Culture Sciences, Department of Physical Culture and Health, University of Szczecin, 70-453 Szczecin, Poland

**Keywords:** Game Ready, combat sport, regeneration, microcirculation, MMA

## Abstract

**Objectives:** This study aimed to evaluate the immediate effect of Game Ready (GR) heat–cold compression contrast therapy (HCCT) on changes in the biomechanical parameters of the quadriceps femoris muscles and tissue perfusion. **Methods:** Fifteen male MMA fighters were subjected to HCCT on the dominant leg’s thigh and control sham therapy on the other. The experimental intervention used a pressure cuff with the following parameters: time—20 min; pressure—25–75 mmHg; and temp.—3–45°C, changing every 2 min. For the control group, the temp. of sham therapy was 15–36 °C, and pressure was 15–25 mmHg, changing every 2 min. Measurements were taken on the head of the rectus femoris muscle (RF) 5 min before therapy, 5 min after, and 1 h after therapy in the same order in all participants: microcirculatory response (PU), muscle tension (MT), stiffness (S), flexibility (E), tissue temperature (°C), and pressure pain threshold (PPT). **Results:** The analysis revealed significant differences between the HCCT and sham therapy groups and the measurement time (rest vs. post 5 min and post 1 h) for PU, MT, E, and °C (*p* < 0.00001) (a significant effect of time was found) in response to GR therapy. No significant differences were found for the PPT. **Conclusions:** The results of this study prove that GR HCCT evokes changes in the biomechanical parameters of the RF muscles and perfusion in professional MMA fighters.

## 1. Introduction

Efforts in MMA fights are characterized by periods of very high work intensity and short rest breaks between subsequent rounds [1]. The combat strategy determines the predominance of various types of force during a fight [2]. Also, depending on a fighter’s preferred style, during a fight, there is a lot of isometric muscle work (grappling maneuvers) or dynamic movements emphasizing concentric muscle work (kicks and punches), which characterizes strikers [3,4].

It is proven that dynamic muscle work causes mechanical damage to cell membranes, the activation of inflammatory processes, muscle soreness, and stiffness and affects muscles’ elasticity and tone [2,5,6]. The quadriceps muscles are particularly susceptible to stress during MMA training, especially in the so-called strikers, i.e., those who use their legs and kick particularly often during fights [7,8]. Some authors suggest that the lack of adequate muscle regeneration after exercise, along with anxiety, stress, and sleep disturbances, is one of the most critical factors of injury in combat sports [9]. The scientific literature emphasizes the importance of the properties of leg muscles in combat sports [10]. It has been shown that increasing knee speed and minimizing time off the ground effectively improves the kicking technique. Moreover, the most important thing is the ability to perform quick maneuvers and change direction while moving on the ground. When it comes to striking execution, the lower body also plays a significant role, as the reaction to ground force is generated and transferred through the lower limbs to the upper body, allowing for robust and quick action [11].

MMA fighters have high training routine loads and often suffer pain after exercise. Considering this, it is necessary to evaluate the regenerative methods used to restore normal resting muscle parameters [12,13]. Faster muscle fatigue and higher injury risk can be caused by increased resting values of muscle tone and, connected with them, the increased intramuscular pressure with which the athlete begins exercise [14]. Due to high training loads, muscle strain, and pain, we consider this group adequate for testing the effects of recovery methods. In the scientific literature, we observe an increase in interest in the assessment of the viscoelastic properties of muscles. In physics and engineering, stiffness is defined as the ratio between the applied force and the strain induced in a structure. In biomechanics, the term “elastic stiffness” is used [15]. This term differs from the sometimes-used term “elasticity” and is connected to the dampening effect of a tissue, such as the elasticity of the skin [15]. Passive stiffness is related to myofascial tissue—muscles, fascias, and tendons. For athletes, quantifying stiffness and flexibility is essential for musculoskeletal injuries, improving muscle performance, and planning physical performance [16,17].

Different heat, cold, and contrast therapies in regeneration procedures are common among physiotherapists, coaches, and athletes of multiple sport disciplines, including MMA [18]. Very few studies have assessed the effectiveness of contrast therapy [19,20,21,22]. There are no RCTs; therefore, the parameters and characteristics of highly effective recovery protocols remain unknown. In the scientific literature, there is a gap particularly in the knowledge of assessing biomechanical muscle changes and their impact on tissue perfusion [23,24]. There are some studies which examine the effects of contrast therapy on the muscular system, but this is conducted by using hot and cold baths, suggesting that they positively impact the process of post-exercise recovery [25,26,27]. No study has yet investigated the effect of Game Ready-type contrast therapy on muscle biomechanical parameters in the same individuals on alternate sides of the body.

The effectiveness of contrast water therapy and the satisfaction of coaches and athletes have prompted clinicians to look for new tools for stimulating the muscular system. One of them is Game Ready (GR) (An Avanos Medical, Inc., Company, 5405 Windward Pkwy, Alpharetta, GA, USA). This system can serve as local monotherapy with cold, heat, contrast therapy, or all these stimuli combined with pressure in one session [28,29]. GR therapy is applied to the chosen body area with a pressure cuff, which makes it an easy-to-use, mobile, and time-saving procedure. Pressure is regulated to range from 15 to 75 (mmHg) (2–10 kPa), and the temperature ranges from 3 to 45 °C. Therapy time is regulated from 5 to 30 min [20,24].

Thus, although GR equipment is widely used, no evidence-based regeneration protocols have been developed for different sports disciplines. The scientific literature about the use of GR therapy in MMA fighters is scarce. In identifying this knowledge gap, we formulated the aims of this study.

This study aimed to assess the immediate impact of heat and cold contrast compression therapy (HCCT) on changes in the values of quadriceps femoris muscle biomechanical properties, pressure pain threshold (PPT), and tissue temperature and changes in tissue perfusion in MMA athletes. Immediate effects are defined as the changes observable just directly after the end of the intervention. We formulated a research hypothesis that the usage of HCCT may significantly change these parameters. To examine the effectiveness of HCCT, the following factors were analyzed: muscle pain measured by the PPT; a physiological perfusion parameter along with tissue temperature; and biomechanical muscle parameters such as elasticity, stiffness, and tension.

## 2. Materials and Methods

**Figure 1:**
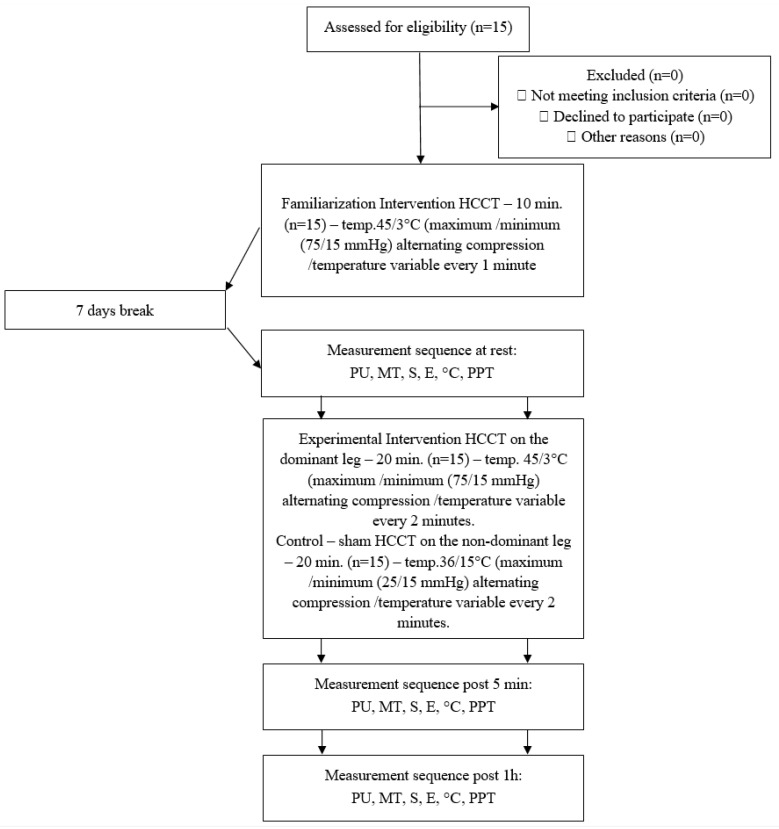
Study design. PU—perfusion; MT—muscle tension; S—stiffness; E—elasticity; °C—tissue temperature; PPT—pressure pain threshold; HCCT—heat–cold compression contrast therapy.

This clinical trial employed a unique, non-randomized, experimental, interventional study design, where the controlled therapy was administered to the same subjects on the opposite side of the body. This innovative approach was accepted by the ethics committee of the polish National Council of Physiotherapists (no. 9/22 of 6 April 2022). This study was registered in the Clinical Trials Register at https://doi.org/10.1186/ISRCTN90040217 and was conducted in accordance with the Declaration of Helsinki. The study design is presented in the flowchart in Figure 1.

### 2.1. Participants

Fifteen male (n = 15) MMA fighters (age: 27.5 ± 5.4 years; training experience: 10.7 ± 5.8, BMI: 25.55 ± 3.0) were enrolled in GR sessions. Interventional GR therapy was applied to the dominant leg’s thigh (right in all cases), and sham therapy was applied to the non-dominant leg’s thigh. In both cases, the time was 20 min (Figure 1). The inclusion criteria were as follows: males aged 18–40 years, MMA training experience of at least 3 years, and training regularly at least 4 times a week. The exclusion criteria were as follows: increased blood pressure (>140/90 mmHg), injuries present or that occurred in the last 6 months before this study in the lower limbs, or any skin abnormalities, wounds, scars, or other in the measurement area. Persons with tattoos on the investigated area of the thighs were excluded as well, since tissue perfusion measurements could possibly be affected. Exclusion criteria included extreme fatigue, fever, or any infection that occurred before or during this study. Participants were informed that they can quit at any time during this study on their explicit request without consequences. All participants were informed about the study conditions, risks, and possibilities, and after that, they signed informed consent forms to participate in this study. Participants were instructed not to perform any training or exercise for 24 h before and during this whole study. Moreover, due to possible effects on tissue perfusion measurements, participants were asked not to consume ergogenic drinks (a list of forbidden drinks was delivered before this study) for twenty-four hours before and during this study. No other nutritional routines of the participants were changed.

### 2.2. Interventions—GR Therapy

**Figure 2:**
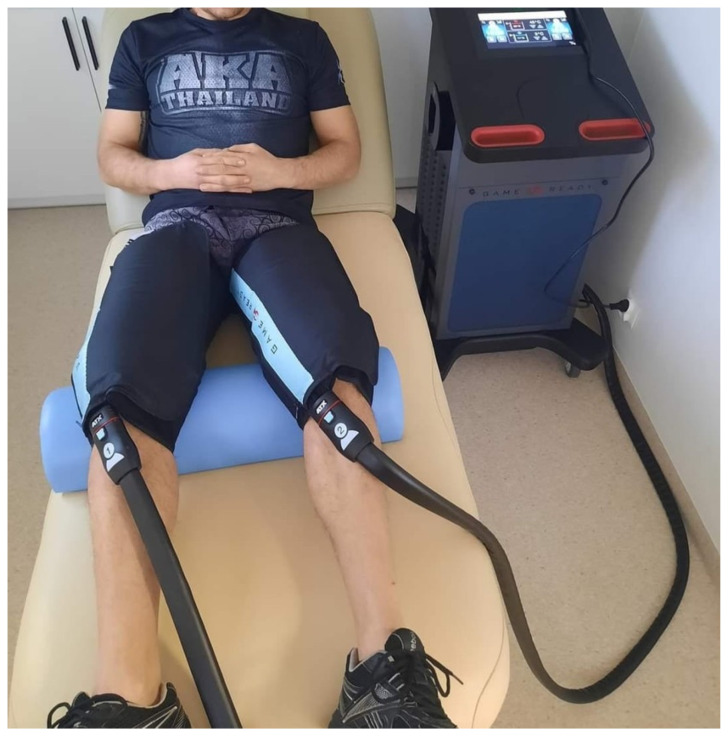
The interventional setup—one of the participants with the cuffs of the Game Ready equipment on his thighs.

Seven days before this study, every participant underwent familiarization with the GR intervention with 10 min stimulation with a cuff applied to the dominant lower limb thigh, performing heat and cold stimulation alternated in a 2 min cycle (Figure 2). The intervention in the experimental site utilized the following parameters: 20 min therapy time, compression pressure values ranging from 25 to 75 (mmHg), and temperature ranging from 3 °C to 45 °C. For the control site—sham therapy—a temperature ranging from 15 °C to 36 °C and pressure ranging from 15 to 25 (mmHg) were used. Our studies, whose results were published before this article, provide evidence that even 5 min of therapy time brings about significant effects. However, we decided to use 20 min of therapy time to strengthen the reliability of the methods and be similar to the typical way of using GR equipment in practice [30,31,32,33]. The GR recovery protocol was developed in the contemporary literature [20,23]. The measurements were performed in the same way and order before therapy—at rest, 5 min after therapy (post 5 min), and 1 h after (post 1 h).

### 2.3. Measurements

**Figure 3:**
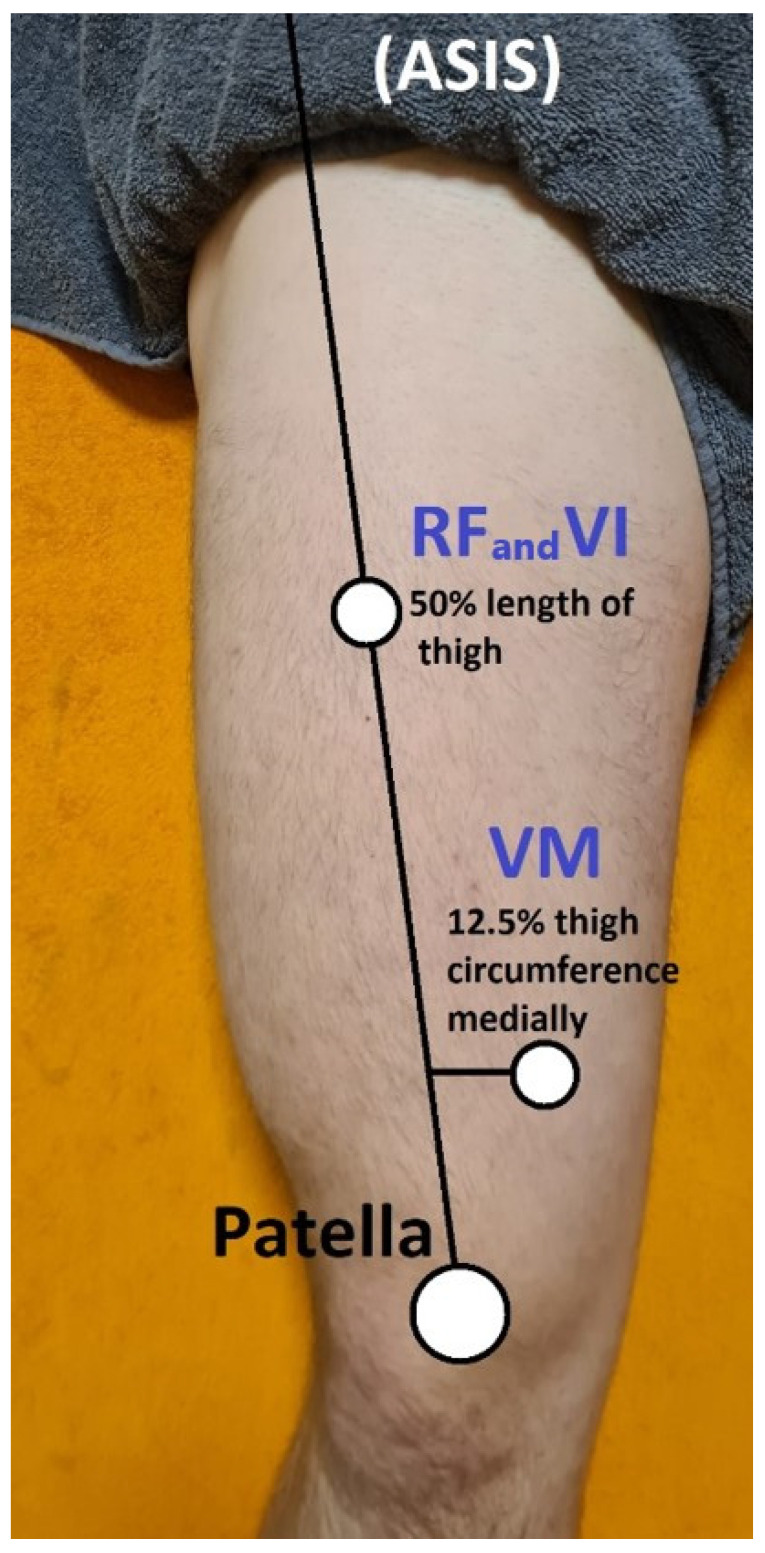
Measurement points. ASIS—Anterior Superior Iliac Spine; RF—rectus femoris; VI—Vastus Intermedius; VM—Vastus Medialis.

During measurements and GR therapy, the participants were in a standardized, relaxed, lying position with a roller under the knee. The measurements at the broadest cross-sectional area of the rectus femoris (RF) muscle were taken with ultrasound-guided equipment (Sonoscape E2, Guangzhou Yueshen Medical Equipment Co., Ltd., Guangzhou, China) in both legs [34] by an experienced professional (Figure 3). The following measurements were recorded for all participants in the RF measurement point which was marked at the 50% length of the distance between the ASIS and patella: microvascular response (PU—[au]), muscle tone (MT—[Hz]), stiffness (S—[N/m]), elasticity (E—[arb—relative arbitrary unit]), tissue temperature (°C), and pressure pain threshold (PPT—[N/cm]). All participants were tested between 9 a.m. and 12 p.m. This research was conducted at the Provita Żory Medical Center, Poland. The measurements were carried out at rest (Rest)—5 min before GR stimulation—and then immediately after GR stimulation at 5 min (Post-GR5 min) and 1 h after GR stimulation (Post-GR1 h). Appropriately trained physiotherapists made the measurements. The order of measurements was as follows in every case and every time point: (1) PU; (2) MT; (3) S; (4) E; (5) °C; and (6) PPT.

#### 2.3.1. Tissue Perfusion—Perfusion Unit (PU)

The Laser Doppler Flowmetry (LDF) is a standard technology for assessing microcirculatory function in vivo [35] and the gold standard, demonstrating great sensitivity and repeatability of measurements [36]. In this study, LDF was used to analyze the PU [37] with Perimed (Perimed AB, Jakobsberg, Sweden) equipment. The measurement point was determined clinically and with the use of USG [25] and then marked with a special pen [37]. The depth of measurement was 2.5 mm, the volume was 1 mm3, and the procedure lasted for two minutes. A precise and easy assessment of the microcirculation at rest and after exertion is possible with the use of LDF due to the non-invasiveness of this method. To assess the microcirculation, the standardized LDF test proposed by Liana et al. was used [38].

#### 2.3.2. Myotonometry

To assess MT, S, and E for the RF, a MyotonPRO myotonometer (Myoton AS, Tallinn, Estonia) was used. This equipment offers digital palpation with an indentation probe of 3 mm diameter. The MyotonPro applies a mechanical impulse to the tissues and records the dynamic tissue response, like displacement and oscillation acceleration signals. Subsequently, the device computes the parameters characterizing MT, S, and E. Its reliability and repeatability have been confirmed in the scientific literature [39,40]. The device evaluates MT based on the oscillation frequency of a muscle [40]. The S assessed by myotonometry characterizes the resistance of the muscle to contraction [41]. Muscle E, defined as the muscle’s ability to regain its original shape after deformation, was measured as a logarithmic decrement in the dampening of tissue oscillations. A faster decrement means a higher dissipation of elastic mechanical energy provoked by the applied impulse. The decrement in natural tissue oscillation inversely describes elasticity [40].

#### 2.3.3. Muscle Pain—Pressure Pain Threshold (PPT)

An algometer (FDIX, Wagner Instruments, Greenwich, CT, USA) served to measure the PPT. Three compression tests with a 4 mm radius probe were performed at the RF measurement point in all participants at every measurement time point. The same person performed the measurements in the same position and conditions every time. The measurer was a professional familiar with using this equipment. The result was calculated as the mean of 3 attempts and displayed on screen. In the case of significant differences in the obtained results, the need to repeat the test was signaled. The pressure force was increased until a stimulus was unpleasant for the participant [42]. Pressure algometers have been widely used in clinical practice for almost one hundred years [43]. This method is widely used to evaluate myofascial pain and different musculoskeletal diseases, demonstrating high reliability [44].

### 2.4. Statistical Methods

Statistica 13.1. (StatSoft Polska, Kraków, Poland) software was used to prepare all statistical analyses. All results are presented as the means with standard deviations. The Shapiro–Wilk and Levene’s tests were utilized to verify the normality of sample data’s variance, homogeneity, and sphericity for a significance analysis of follow-up with two-way functional variance with repeated measures. Effect sizes for main effects and interactions were determined by partial eta-squared (η^2^). Partial eta-squared values were classified as small (0.01 to 0.059), moderate (0.06 to 0.137), and large (>0.137). Post hoc comparisons were performed with the Tukey test to locate differences between mean values when a main effect or interaction was found. Percentage changes with 95% confidence intervals (95 CIs) were also calculated. Statistical significance was assumed at the level of *p* < 0.05. The G*Power program performed an a priori power analysis [45]. A repeated measures ANOVA between interactions with an effect size of at least 0.25, α = 0.05, and 1 − β = 0.95 yielded a statistical power of 97.37% and a minimum sample size of 15 subjects.

## 3. Results

The results of the variance analysis are presented in Table 1. The analysis revealed significant differences between the groups and time (Rest vs. post 5 min and post 1 h) for PU, MT, E, °C, and the PPT in response to GR therapy (a significant effect the time was found).

**Figure 4:**
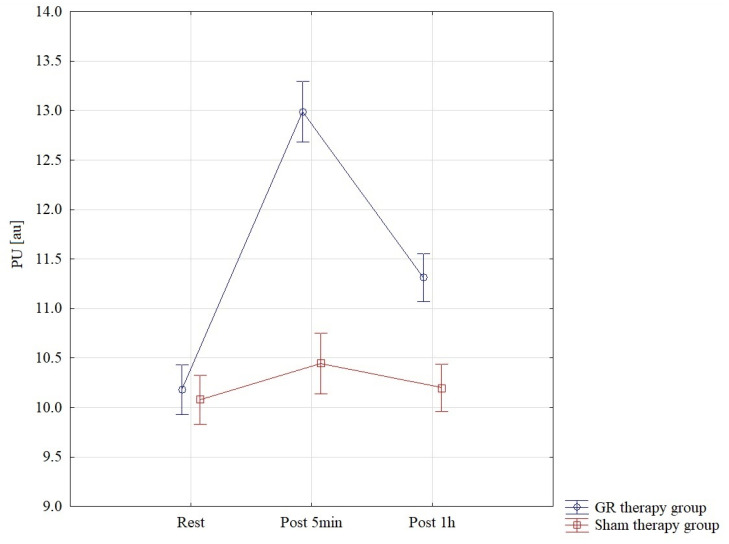
Comparison of mean values of confidence intervals by group (GR therapy and sham therapy) for perfusion—PU: F(2, 56) = 67.69, *p* < 0.001.

The results of the analysis of variance for PU (perfusion unit) allowed for the finding of significant differences for the pre–post*therapy interaction F = 67.70; *p* < 0.001; η^2^ = 0.71. Significant differences were found between all interactions *p* < 0.001 in the GR therapy group. The GR therapy group found the highest PU results, measured 5 min after therapy, M = 12.99 ± 0.63, and they were statistically significantly higher than those in the sham therapy group at 5 min of therapy M = 10.45 ± 0.53. In the GR group, PU one hour after therapy, M = 11.31 ± 0.45, was statistically significantly higher than that in the sham therapy group M = 10.20 ± 0.46 *p* < 0.001. These results are also confirmed by Figure 4.

**Figure 5:**
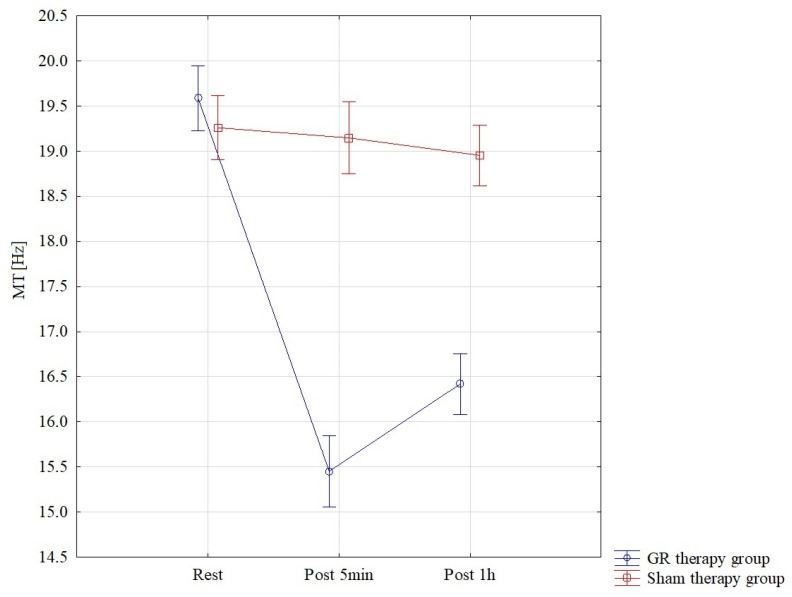
Comparison of mean values of confidence intervals by group (GR therapy and sham therapy) for muscle tension—MT: F(2, 56) = 94.57, *p* < 0.001.

The results of the analysis of variance for MT (muscle tension) allowed for the finding of significant differences for the pre–post*therapy interaction F = 94.57; *p* < 0.001; η^2^ = 0.77. Significant differences were found between all interactions *p* < 0.001 in the GR therapy group. The lowest MT results were found in the GR therapy group, measured 5 min after therapy, M = 15.45 ± 0.93, and they were statistically significantly lower than those in the sham therapy group in the 5th minute of therapy, M = 19.15 ± 0.51. In the GR group, MT one hour after treatment M = 16.42 ± 0.71 was statistically significantly lower than that in the sham therapy group M = 18.95 ± 0.55 *p* < 0.001. These results are also confirmed by Figure 5.

**Figure 6:**
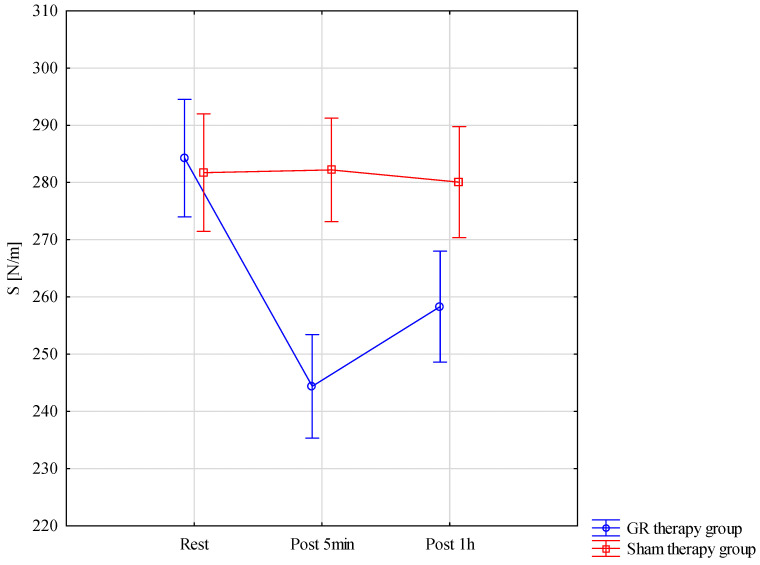
Comparison of mean values of confidence intervals by group (GR therapy and sham therapy) for stiffness—S: F(2, 56) = 72.23, *p* < 0.001.

The results of the analysis of variance for S (stiffness) were divided into detailed criteria for pre–post*therapy applications F = 72.23; *p* < 0.001; η^2^ = 0.72. The risk of interaction between disturbances in the Gr group was *p* < 0.001. The lowest results for S occurrence in the GR therapy group measured 5 min after treatment was M = 244.4 ± 14.78, with there being one statistically significant difference than in the sham therapy group 5 min after treatment M = 282.2 ± 19.14. In the GR group, S one hour after therapy, M = 258.33 ± 18.43, was statistically significantly more effective than in the sham therapy group M = 280 ± 18.20 *p* < 0.001. These results are also confirmed by Figure 6.

**Figure 7:**
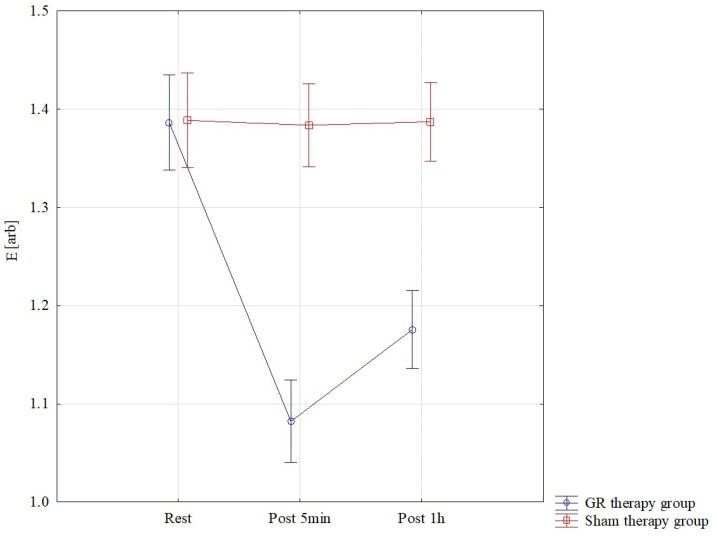
Comparison of mean values of confidence intervals by group (GR therapy and sham therapy) for elasticity—E: F(2, 52) = 77.88, *p* < 0.001.

The results of the analysis of variance for E (elasticity) allowed for the finding of significant differences for the pre–post*therapy interaction F = 77.88; *p* < 0.001; η^2^ = 0.74. Significant differences were found in the GR therapy group between all interactions, *p* < 0.001. The lowest E scores were found in the GR therapy group measured 5 min after therapy, M = 1.08 ± 0.05, and they were statistically significantly lower than those in the sham therapy group 5 min after treatment, M = 1.38 ± 0.09. In the GR group, E therapy one hour after treatment M = 1.18 ± 0.05 was statistically significantly lower than that in the sham therapy group M = 1.39 ± 0.09 *p* < 0.001. These results are also confirmed by Figure 7.

**Figure 8:**
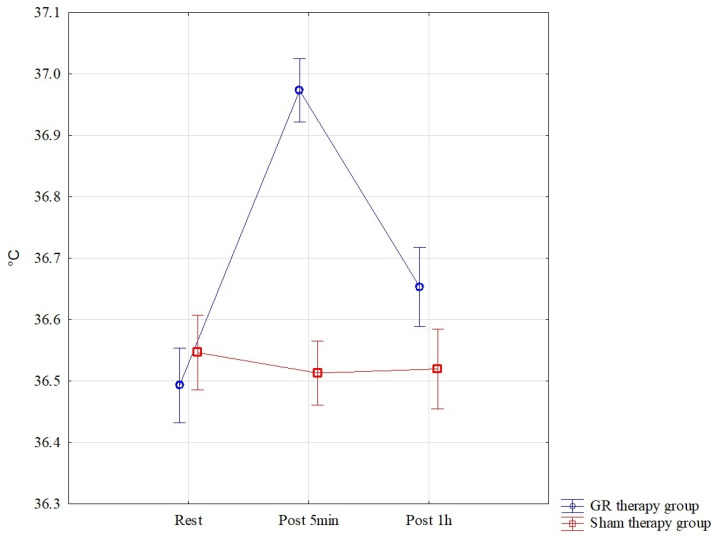
Comparison of mean values of confidence intervals by group (GR therapy and sham therapy) for temperature—°C: F(2, 56) = 45.83, *p* < 0.001.

The results of the analysis of variance for °C (tissue temperature) allowed for the finding of significant differences for the pre–post*therapy interaction F = 45.83; *p* < 0.001; η^2^ = 0.62. Significant differences were found between all interactions *p* < 0.001 in the GR therapy group. The highest °C results were found in the GR therapy group measured 5 min after therapy, M = 36.97 ± 0.11, and they were statistically significantly higher than those in the sham therapy group 5 min after treatment, M = 36.51 ± 0.08. In the GR group, °C therapy, one hour after treatment, M = 36.65 ± 0.14, was statistically significantly higher than that in the sham therapy group, M = 36.52 ± 0.10 *p* = 0.024. These results are also confirmed by Figure 8.

**Figure 9:**
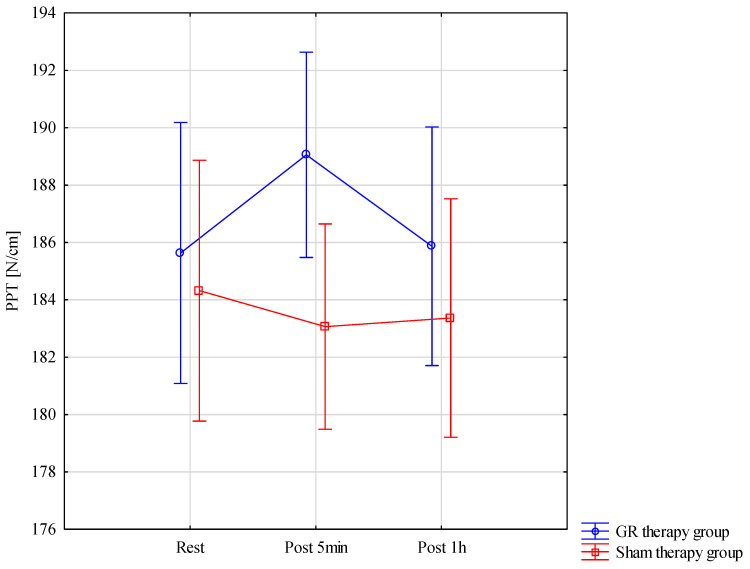
Comparison of mean values of confidence intervals by group (GR therapy and sham therapy) for pressure pain threshold—PPT: F(2, 56) = 2.94, *p* = 0.061.

The results of the analysis of variance for the PPT (pressure pain threshold) did not allow for the finding of significant differences for the pre–post*therapy interaction F = 2.94; *p* = 0.061; η^2^ = 0.095. These results are also confirmed by Figure 9.

## 4. Discussion

This study’s primary objective was to evaluate GR HCCT’s acute effects on the biomechanical parameters of muscles: stiffness, tone, elasticity, and perfusion unit. In the case of the pressure pain threshold, differences between the experimental and control site did not show any statistical significance. The main findings confirm the impact of GR therapy on the reduction in resting muscle tension and stiffness and on increased tissue perfusion, especially in flow-dependent dilation (FMD). These changes play an essential role for the adaptive capacity of the vascular endothelium, which in turn affects the processes of post-exercise regeneration significantly [46,47].

### 4.1. Tissue Perfusion

Skeletal muscle contains a dense network of capillaries that supply nutrients and remove waste products of metabolism and heat from skeletal muscle cells [48]. This is essential during exercise, during which the metabolic rate is dramatically increased with a dependent increase in the supply of oxygen, which is the recipient through blood flow and the recruitment of capillary units, characterized by increased flow and red blood cells [49]. The assessment of microcirculation function is also essential in the regenerative process. In the scientific literature, there is a hypothesis that tissue perfusion reflects the functional response of the microcirculation. Different interventions, such as cold or heat stimuli, can elicit various hemodynamic responses and bring improvement in these physiological capacities [50,51,52]. Changes in FMD determine the adaptive capacity of the vascular endothelium, which is reflected in the regeneration processes after exercise [47]. The increased blood flow as measured by LDF postulate that hyperemic responses facilitate the recovery process by promoting nutrient delivery. This study demonstrated a significant improvement in applying HCCT to tissue perfusion. Some authors suggest that PU measured by LDF may possibly have a counterpart in the responses of the muscles [53]. Akasaki et al. showed in an experiment on animals that repeated thermal therapy had increased blood flow, capillary density, and endothelial nitric oxide synthase (eNOS) protein expression in the ischemic hindlimb of mice [54]. The endothelium as a source of numerous mediators is proven to play a crucial role in the mechanism of local microvascular autoregulation, with the strongest vasodilators being nitric oxide (NO) and prostacyclin and the strongest vasoconstrictors being EDCF1 and EDCF2 [55].

### 4.2. Biomechanical Properties

Muscle tension, stiffness, and elasticity are maintained generally by the complex interaction of spinal and supraspinal mechanisms, whose disruption brings about changes in the biomechanical properties of muscles [56]. Thermal stimulus, which causes an increase in muscle congestion, reduces muscle tension and improves muscle stiffness and elasticity [52]. The authors of this study have similar observations. HCCT reduced tissue tension and stiffness, increasing elasticity, which correlated with increased tissue perfusion. Although the mechanisms causing this effect are still not clear, the non-myogenic regulation of muscle tone associated with increased perfusion is accepted in the scientific literature. In many sports disciplines, specific levels of muscle elasticity and stiffness are needed. The optimal level and relation of these parameters differ in many sports (i.e., swimming vs. sprint running), but no matter what sport, fatigue influences these parameters. The recovery process aims to make the return of these parameters to the baseline effective. Impaired perfusion causes hypoxia and elevated Ca^2+^ levels in the cytosol, which in turn cause muscle contraction due to the activation of the phosphorylation of myosin light chains and the subsequent cross-bridging of actomyosin, which finally generates an increase in muscle tone. Muscle tone can be reduced by the activation of the capillary system which eliminates subclinical manifestations of tissue hypoxia [51].

Stiffness, muscle tension, and elasticity were the subjects of the authors’ research as parameters that have an essential role in the muscles’ strength and power production and, moreover, may influence the risk of injury in sports [57]. Evidence suggests that a more flexible (and less stiff) musculotendinous system has a more remarkable ability to be lengthened, optimizing the absorption of external forces and exerting a ‘moderating effect on energy production during function’ [46]. Muscles dissipate energy during active lengthening, and energy dissipation affects a wide range of locomotor activities with a predominance of excentric function. Injuries occur during these activities [58].

### 4.3. Muscle Pain

Muscle pain is initiated by activating nociceptors through thermal, mechanical, and chemical actions [59]. In MMA, we often deal with mechanical stimuli, such as exercises and injuries, in the form of hits [60]. Due to the specificity of this physical activity, the threshold for practicing MMA should be considered. However, MMA can still transmit disrupted movement patterns, impacting injury risk and athletic performance. Therefore, assessing the pain threshold for MMA fighters is essential [18].

Although our PPT results do not confirm an analgesic effect, this does not mean that GR therapy does not affect muscle pain. A general hypothesis about the analgesic effect of contrast therapy is emphasized in the scientific literature [27]. The effectiveness of the use of GR therapy in the treatment of muscle pain has insufficient evidence in the scientific literature. Many research projects focused on using GR therapy in connection with pain changes in sports injuries [23] and the use of other forms of contrast therapy with the domination of water therapy and compresses [26,61,62]. There are many studies showing that water contrast therapy reduces the adverse effects of exercise-associated muscle damage (EAMD), inflammation, and delayed-onset muscle soreness (DOMS) [63], simultaneously increasing the process of muscle strength recovery [27], power, and joint mobility after debilitating exercise [64]. Colantuono et al. noted that former studies of contrast compression therapy did not investigate muscle recovery capacity after intense efforts or conduct an evaluation of the recovery of intramuscular glycogen stores. There are results suggesting a positive effect of HCCT on the recovery process and recovery of intramuscular glycogen stores associated with EAMD after intensive eccentric exercise [27].

### 4.4. Limitations

The limitation of this research is that the results only indicate an immediate effect. Undoubtedly, follow-ups should be extended in subsequent studies. Another limitation can be the fact that part of the measured values do not have the reference values described. Additionally, the LDF method and myotonometry are treated as susceptible by some. Even though, the LDF [65] and myotonometry [66] methods are sensitive ones; current scientific knowledge proves they are well validated, reliable, and widely used. Considering this, the authors used the assessment of changes in response to a stimulus (in this case, GR) instead. Using the other side of the same participant as a control can be interpreted as a limitation. However, this brings some benefits—the measurements can be made at the same time points on both sides—experimental and control. We can observe changes on the experimental side, having as a point of reference tissues that are as similar as possible to those on the experimental side. Only 15 male MMA fighters took part in this study. Recruiting entirely professional MMA fighters at the same sporting level was associated with many difficulties. A multicenter crossover study with three separate, randomized groups is planned in subsequent protocols. Additionally, it may be interesting to observe changes in the stiffness, resting tension, or flexibility evoked by GR therapy in comparison with other methods of recovery. Future projects are recommended to focus on the assessment and comparison of the effects of GR therapy in athletes of different levels and sport disciplines, as well as after the extreme fatigue of different characteristics.

## 5. Conclusions

This study provides evidence that HCCT performed with GR causes an immediate effect after therapy and is an effective method that influences muscle biomechanical parameters like the stiffness, elasticity, tone, and tissue perfusion of the quadriceps femoris muscle in MMA athletes. Since the measurements were performed at rest, there was no significant impact on the muscle pain measured by the pressure pain threshold with an algometer. The use of HCCT needs more research aimed at designing the most effective recovery protocols in different sports disciplines.

## Figures and Tables

**Table 1:** Comparison between GR and sham therapy groups for all measured variables.

	Measurement Time	GR Therapy Group				Sham Therapy Group			
		M	SD	−95%CI	+95%CI	M	SD	−95%CI	+95%CI
PU [au]	Rest	10.18	0.59	9.85	10.51	10.08	0.31	9.91	10.25
	Post 5 min	12.99	0.63	12.64	13.33	10.45	0.53	10.15	10.74
	Post 1 h	11.31	0.45	11.07	11.56	10.20	0.46	9.94	10.46
MT [Hz]	Rest	19.59	0.75	19.17	20.00	19.26	0.59	18.94	19.58
	Post 5 min	15.45	0.93	14.94	15.97	19.15	0.51	18.86	19.43
	Post 1 h	16.42	0.71	16.03	16.81	18.95	0.55	18.65	19.26
S [N/m]	Rest	284.27	21.45	272.39	296.15	281.73	17.17	272.22	291.24
	Post 5 min	244.40	14.78	236.22	252.58	282.20	19.14	271.60	292.80
	Post 1 h	258.33	18.43	248.12	268.54	280.07	18.20	269.99	290.14
E [arb]	Rest	1.39	0.09	1.34	1.44	1.39	0.09	1.34	1.44
	Post 5 min	1.08	0.05	1.05	1.11	1.38	0.09	1.33	1.44
	Post 1 h	1.18	0.05	1.15	1.20	1.39	0.09	1.33	1.44
Temp. [°C]	Rest	36.49	0.12	36.43	36.56	36.55	0.11	36.49	36.61
	Post 5 min	36.97	0.11	36.91	37.03	36.51	0.08	36.47	36.56
	Post 1 h	36.65	0.14	36.58	36.73	36.52	0.10	36.46	36.58
PPT [N/cm]	Rest	185.63	8.62	180.86	190.41	184.32	8.57	179.57	189.07
	Post 5 min	189.05	6.01	185.72	192.38	183.07	7.44	178.94	187.19
	Post 1 h	185.87	6.61	182.20	189.53	183.37	8.94	178.42	188.32

Variables: blood perfusion (PU), muscle tension (MT), dynamic stiffness (S), elasticity (E), tissue temperature (°C), pressure pain threshold (PPT).

## Data Availability

Data are available from the corresponding author only at the justified request of a scientist.

## Abbreviations

BMI	body mass index
CGs	compression garments
HCCT	heat and cold contrast compression therapy
DOMS	delayed-onset muscle soreness
E	elasticity
EAMD	exercise-associated muscle damage
EDCF1	endothelium-derived contracting factor-1
EDCF2	endothelium-derived contracting factor-2
EMG	electromyography
eNOS	endothelial nitric oxide synthase
FMD	flow-mediated dilation
GR	Game Ready
HSP	heat shock protein
LDF	laser Doppler flowmeter
MMA	mixed martial arts
NO	nitric oxide
PPT	pressure pain threshold
PU	perfusion unit
S	stiffness
MT	muscle tone
